# Polymorphisms in the vascular endothelial growth factor gene and breast cancer in the Cancer Prevention Study II cohort

**DOI:** 10.1186/bcr1400

**Published:** 2006-04-13

**Authors:** Eric J Jacobs, Heather Spencer Feigelson, Elizabeth B Bain, Kerri A Brady, Carmen Rodriguez, Victoria L Stevens, Alpa V Patel, Michael J Thun, Eugenia E Calle

**Affiliations:** 1 Department of Epidemiology and Surveillance Research, American Cancer Society, Atlanta, Georgia, USA

## Abstract

**Introduction:**

Vascular endothelial growth factor (VEGF) plays a central role in promoting angiogenesis and is over-expressed in breast cancer. At least four polymorphisms in the *VEGF *gene have been associated with changes in VEGF expression levels: -2578C/A, -1154G/A and -634G/C are all located in the promoter region; and +936C/T is located in the 3'-untranslated region.

**Method:**

We examined the association between these four *VEGF *polymorphisms and risk for breast cancer among postmenopausal women in CPS-II (Cancer Prevention Study II) Nutrition Cohort. This cohort was established in 1992 and participants were invited to provide a blood sample between 1998 and 2001. Included in this analysis were 501 postmenopausal women who provided a blood sample and were diagnosed with breast cancer between 1992 and 2001 (cases). Control individuals were 504 cancer-free postmenopausal women matched to the cases with respect to age, race/ethnicity, and date of blood collection (controls).

**Results:**

We found no association between any of the polymorphisms examined and overall breast cancer risk. However, associations were markedly different in separate analyses of invasive cancer (*n *= 380) and *in situ *cancer (*n *= 107). The -2578C and -1154G alleles, which are both hypothesized to increase expression of VEGF, were associated with increased risk for invasive breast cancer (odds ratio [OR] 1.46, 95% confidence interval [CI] 1.00–2.14 for -2578 CC versus AA; OR 1.64, 95% CI 1.02–2.64 for -1154 GG versus AA) but they were not associated with risk for *in situ *cancer. The +936C allele, which is also hypothesized to increase VEGF expression, was not clearly associated with invasive breast cancer (OR 1.21, 95% CI 0.88–1.67 for +936 CC versus TT/CT), but it was associated with reduced risk for *in situ *cancer (OR 0.59, 95% CI 0.37–0.93 for CC versus TT/CT). The -634 C/G polymorphism was not associated with either invasive or *in situ *cancer.

**Conclusion:**

Our findings provide limited support for the hypothesis that the -2578C and -1154G *VEGF *alleles are associated with increased risk for invasive but not *in situ *breast cancer in postmenopausal women.

## Introduction

Angiogenesis, the development of new blood vessels, is required for the growth of microscopic cancers into larger, clinically relevant tumors [1]. Vascular endothelial growth factor (VEGF) is believed to play a central role in angiogenesis through a variety of mechanisms, including effects on endothelial cell proliferation, survival, and migration [2]. VEGF is over-expressed even in early stage breast cancers [3], and high tumor VEGF expression is associated with both increased tumor microvessel density and increased risk for breast cancer recurrence [4-7].

The *VEGF *gene includes at least four polymorphisms that are relatively common and may influence VEGF expression. Three of these polymorphisms are located in the promoter region at -2578C/A (rs699947), -1154G/A (rs1570360) and -634G/C (rs2010963), relative to the translation start site. The -2578C, -1154G and -634C alleles have all been associated with increased VEGF expression [8,9]. The -1154G allele has also been associated with melanoma thickness [10], prostate cancer incidence and progression [11], and high VEGF expression within lung tumors [12]. In addition to these promoter region polymorphisms, the C allele of a common polymorphism located in the 3' untranslated region (+936C/T; rs3025039) is associated with substantially increased serum VEGF levels [13,14].

To our knowledge, three previous studies [14-16] have examined the association between *VEGF *polymorphisms and breast cancer risk. An Austrian study [14], including 500 cases and 500 controls, found the +936C allele to be associated with substantially increased risk for breast cancer (odds ratio [OR] 2.0, 95% confidence interval [CI] 1.4–2.6). A smaller study from the UK [15], including 134 cases and 263 controls, found no statistically significant association between the -1154G/A polymorphism and breast cancer risk. Recently, results were published from a case-control study including 571 familial breast cancer cases from Poland and Germany and 974 unselected breast cancer cases from Sweden [16]. That study examined both the three promoter region polymorphisms (-2578C/A, -1154G/A, and -634G/C) and the +936C/T polymorphism. There was no suggestion of any association between any of these polymorphisms and breast cancer risk, although -1154G/A was examined using only the familial cases and -634G/C was examined using only the unselected Swedish cases. However, there was some evidence that the promoter region polymorphisms might be associated with cancer progression. In analyses conducted among cancer cases, the -2578C allele was associated with significantly higher histologic grade, and the -634C allele was associated with both significantly higher histologic grade and increased tumor size.

To explore further the role played by potentially functional *VEGF *polymorphisms, we examined the association between the three promoter region polymorphisms (-2578C/A, -1154G/A and -634G/C) and a 3'-untranslated region polymorphism (+936C/T) and risk for incident breast cancer using postmenopausal cases and controls from the CPS-II (Cancer Prevention Study II) Nutrition Cohort.

## Materials and methods

### Study population

Women included in the analysis were participants in the CPS-II Nutrition Cohort [17]; the CPS-II is a prospective study of cancer incidence including approximately 184,000 US men and women, established by the American Cancer Society. At enrollment into the Nutrition Cohort in 1992 or 1993, all participants completed a self-administered questionnaire that included questions on demographics, medical and reproductive history, and lifestyle factors. Most participants were age 50–74 years at the time of enrollment. Beginning in 1997, follow-up questionnaires were sent to cohort members every 2 years to update exposure information and to ascertain newly diagnosed cancers. Incident cancers reported on questionnaires were verified through medical records, linkage with state cancer registries, or death certificates. The recruitment, characteristics, and follow up of the CPS-II Nutrition Cohort are described in detail elsewhere [17].

From June 1998 through June 2001, participants in the CPS-II Nutrition Cohort were invited to provide a blood sample. After obtaining informed consent, blood samples were collected from 39,371 participants, including 21,963 women. Among postmenopausal women who had provided a blood sample, we initially identified 509 women who had been diagnosed with breast cancer between 1992 and 2001 and had not been diagnosed with any other cancer (other than nonmelanoma skin cancer; cases). Most of these cases (*n *= 328) were diagnosed before they provided a blood sample. Sixty-four per cent of these prevalent cases provided a blood sample within 2 years of their diagnosis date, although the maximum time between diagnosis and blood draw was 7.6 years. For each case, we randomly selected one control from among the postmenopausal women who had provided a blood sample and were cancer free at the time of the case diagnosis. Each control was individually matched to a case with respect to birth date (± 6 months), date of blood collection (± 6 months), and race/ethnicity (white, African-American, Hispanic, Asian, other/unknown). Eight of the cases and five of the controls initially selected were later excluded because genotyping failed for all polymorphisms examined, because on more detailed review they were found not to have been truly postmenopausal or not to have had breast cancer (if a case), or if no DNA sample was available. A total of 501 cases and 504 controls remained for analysis.

### Laboratory methods

DNA was extracted from buffy coat and genotyping assays were performed using TaqMan (Applied Biosystems, Foster City, CA, USA). Genotyping was performed by laboratory personnel blinded to case-control status and 10% blind duplicates were randomly interspersed with the case-control samples for quality control. Concordance for these quality control samples was 100%. The genotyping success rate was 95% or greater for each of the polymorphisms examined. The genotype distribution among controls was in Hardy-Weinberg equilibrium (*P *> 0.05) for all of the polymorphisms examined.

### Statistical analysis

In preliminary analyses using only matched pairs, results were similar when we used a matched pair analysis and when we adjusted for the matching variables. To make use of information from all genotyped cases and controls, we therefore calculated ORs using a conditional logistic regression model that adjusted for each of the matching variables rather than using a matched pair analysis. All models are adjusted for birth year (in single year categories), blood collection date (in single year categories), and race/ethnicity (white, African-American, Hispanic, Asian, or other/unknown).

We examined the association between breast cancer risk and each *VEGF *polymorphism (-2578C/A, -1154G/A, -634G/C, and +936C/T). To simplify interpretation of results, we used the low expression allele of each polymorphism as the referent group when calculating ORs. Because the T allele of the +936C/T polymorphism was uncommon (allele frequency of 14% among controls), we combined TT and CT genotypes when calculating ORs. *P *values for trend were calculated using the number of high VEGF expression alleles (0, 1, or 2). We also examined an additional polymorphism, -1497T/C (rs833061), but found this analysis to be uninformative because alleles for -1497T/C were almost perfectly correlated with those for -2578C/A, for which there is more evidence of functional importance.

Because we hypothesized that combined effects of *VEGF *polymorphisms might be important, we examined the association between breast cancer risk and a VEGF 'blood prediction score', that we created *a priori *(before any genotyping results were seen). The +936C allele has been associated with increased VEGF blood levels [13,14]. In addition, individuals with promoter region haplotypes that included the -2578C allele appeared to have substantially increased blood VEGF levels in one study [8], although analyses of individual polymorphisms were not presented. We therefore defined each individual's blood prediction score as the sum of their +936C and -2578C alleles.

Because promoter region polymorphisms in the *VEGF *gene are in linkage disequilibrium [8], we examined associations between breast cancer risk and haplotypes defined by the three promoter region polymorphisms (-2578C/A, -1154G/A, and -634G/C). Haplotype frequencies were estimated using the partition-ligation expectation-maximization algorithm implemented in the program tagSNPS [18]. Estimated haplotype frequencies were all within 0.005 of an integer value (0, 1, or 2), and so we rounded the estimated haplotype frequencies to the nearest integer. We tested the global hypothesis of no association between the measured promoter haplotypes and breast cancer risk using a likelihood ratio test comparing a model that included variables for each haplotype with a model that did not include these variables. We also examined associations between breast cancer risk and promoter diplotypes, defined by the combination of the two haplotypes carried by each participant.

## Results

Breast cancer cases and controls in this analysis were predominantly white (approximately 98% of both cases and controls) and middle aged or elderly at time of diagnosis (median age 68 years). Table 1 shows the association between each individual *VEGF *polymorphism and risk for breast cancer. Because we hypothesized that *VEGF *polymorphisms may play a role in breast cancer progression, results are shown separately for *in situ *breast cancer and invasive breast cancer, as well as for all breast cancers combined. None of the polymorphisms were statistically significantly associated with all breast cancers combined. However, results differed markedly for *in situ *and invasive breast cancer. For *in situ *breast cancer, the high expression alleles of all four polymorphisms were associated with ORs below 1, although only the +936C allele was associated with a statistically significant reduction in risk (OR 0.59, 95% CI 0.37–0.93 for +936 CC versus CT/TT). In contrast, for invasive breast cancer ORs were above 1 for the high expression alleles of all four polymorphisms, although only the -2578C and -1154G alleles were associated with statistically significant increases in risk (OR 1.46, 95% CI 1.00–2.14 for -2578 CC versus AA; OR 1.64, 95% CI 1.02–2.64 for -1154 GG versus AA). These associations did not appear to differ for regional or distant breast cancers compared with only locally invasive breast cancers (results not shown), although there were few regional or distant cases (*n *= 71). Results did not appear to differ by tumor size or grade (results not shown), although information on tumor size was available for only 38% of *in situ *cancer cases. Results did not change meaningfully when we adjusted for breast cancer risk factors in this study population, including use of hormone replacement therapy, history of breast cysts, family history of breast cancer, parity, and adult weight gain.

Table 1 also shows results for the blood prediction score, an *a priori *measure that combined information from two polymorphisms (-2578C/A and +936C/T) that are associated with VEGF blood levels (see Materials and method, above). The blood prediction score was not associated with risk for all breast cancers combined. However, the blood prediction score was associated with both significantly reduced risk for *in situ *breast cancer (OR 0.73, 95% CI 0.57–0.96 per 1 point increase in the blood prediction score) and significantly increased risk for invasive breast cancer (OR 1.20, 95% CI 1.02–1.41 per 1 point increase in blood prediction score).

The three promoter region polymorphisms we examined are in linkage disequilibrium [8]. We therefore examined haplotypes defined by these three polymorphisms (-2578/-1154/-634; Table 2). Only four haplotypes were identified, the same haplotypes that were reported in a previous study [8]. Haplotype results are shown only for invasive breast cancer because associations between individual polymorphisms appeared to be different for invasive and *in situ *cancer, and there were relatively few *in situ *cases (*n *= 107). The AAG haplotype, the only haplotype that did not include the 1154G allele, was associated with reduced risk (OR 0.62, 95% CI 0.38–1.00 for two copies versus none). The global test of association between measured promoter haplotypes and breast cancer risk (see Materials and method, above) yielded a *P *value of 0.04. There was a moderate degree of correlation between the three promoter region polymorphisms. Among controls in our study, *r*^2 ^(correlation coefficient squared) was 0.55 for -2578C/A and -1154G/A, 0.49 for -2578C/A and -634G/C, and 0.27 for -1154G/A and -634G/C. Among controls in our study, the linkage disequilibrium coefficient (D') was 0.99 for -2578C/A and -1154G/A, 1.00 for -2578C/A and -634G/C, and 1.00 for -1154G/A and -634G/C.

In addition to examining haplotypes, we also examined the association between *VEGF *promoter diplotypes and risk for invasive breast cancer (results not shown). No diplotype was statistically significantly associated with risk for invasive breast cancer, although ORs were greater than 1.4 for each of the six diplotypes that included two copies of the -1154G allele compared with the AAG/AAG diplotype (the diplotype hypothesized to have the lowest expression and used as the referent group).

## Discussion

In this study, alleles of two promoter region polymorphisms hypothesized to increase VEGF expression and a combined measure of high VEGF expression alleles (the blood prediction score) were associated with increased risk for invasive breast cancer. In contrast, the blood prediction score was associated with reduced risk for *in situ *breast cancer. Although these results could be due to chance, it is biologically plausible that increased VEGF expression could accelerate the progression from *in situ *to invasive breast cancer. Whereas *in situ *breast cancers are by definition confined to narrow ducts and may not require angiogenesis, the development of tumors beyond 1–2 mm in diameter is believed to require angiogenesis [1].

When interpreting results it should be noted that the -2578C/A and -1154G/C polymorphisms that were associated with increased risk for invasive breast cancer are in linkage disequilibrium. Therefore, the associations of these two polymorphisms with breast cancer risk are not statistically independent. In addition, the AAG promoter haplotype, which was associated with significantly reduced risk for invasive cancer, was the only haplotype that did not include the -1154G allele. Therefore the reduced risk associated with the AAG promoter haplotype and the increased risk associated with -1154G allele are simply different ways to describe the same underlying association.

Our results differ from those of the largest study of *VEGF *polymorphisms and breast cancer to date, a recent European study that included both unselected cases of breast cancer from Sweden, and a separate group of familial breast cancer cases, predominantly from Poland [19]. This European study examined the same four *VEGF *polymorphisms that we did, but they found no associations with breast cancer risk. The difference in results between our study and the European study could be due to chance. Alternatively, there are several notable differences between the types of breast cancer cases included that might have contributed to the differences in results. First, in the European study only familial breast cancer cases were used to examine the -1154G/A polymorphism, which is the *VEGF *polymorphism most strongly associated with invasive cancer in our study. Second, the European study probably included a substantial number of premenopausal breast cancer cases (the median age of the unselected Swedish cases at diagnosis was 54 years), whereas our study included only postmenopausal cases. Finally, in populations in which detection of *in situ *breast cancer is common, women carrying high expression *VEGF *alleles could be at increased risk for invasive cancer because their breast cancers may be more likely to progress rapidly enough to escape detection as *in situ *cancer. This mechanism could have played an important role in our study, in which approximately 20% of breast cancer cases were diagnosed as *in situ *cancers (presumably due to the high prevalence of mammographic screening [20]), but it is unlikely to have been important in the European study population, in which detection of *in situ *breast cancers appears to have been rare [19].

With respect to other previous studies, we did not observe the increased risk for invasive cancer associated with the +936C allele that was observed in an Austrian study [14]. The association between the -1154G allele and breast cancer risk observed in our study was not observed in a previous study from the UK [15], although the UK study included only 134 breast cancer cases and had limited statistical power.

Because most breast cancer cases included in this analysis were prevalent (diagnosed before the time of blood collection), it is important to consider the potential for survival bias. Women with breast cancer in this cohort who did not survive until the time when blood samples were collected could not be included in the study. Therefore, if pro-angiogenic *VEGF *alleles are associated with reduced breast cancer survival, we may have underestimated their effect on breast cancer incidence. A recent Chinese study [21] reported that the -634G allele was associated with reduced overall survival among breast cancer patients, although there was no association with breast cancer recurrence. However, important survival bias appears unlikely in our cohort, given that survival rates from breast cancer were quite high. Only 5% of breast cancer cases diagnosed in the CPS-II Nutrition Cohort during the years included in this analysis died from breast cancer before we completed blood collection in the cohort. The two largest previous studies of *VEGF *polymorphisms and breast cancer [15,19] also included prevalent breast cancer cases, and the extent to which survival bias might have affected their results is unclear.

The main limitation of the present study is that it is only of moderate size. Larger studies will be needed to examine potentially important differences by stage more thoroughly. Strengths of the study include the potential functional importance of the polymorphisms examined and the fact that we were able to examine common haplotypes in the *VEGF *promoter region. To our knowledge, this is the first study to examine specifically the association between *VEGF *polymorphisms and *in situ *breast cancer, although the number of *in situ *cancers in our study was relatively small.

## Conclusion

Our findings provide limited support for the hypothesis that the -2578C and -1154G *VEGF *alleles are associated with increased risk for invasive breast cancer in postmenopausal women. Any future studies of the association between *VEGF *polymorphisms and breast cancer risk should examine whether results differ for *in situ *and invasive breast cancers.

## Abbreviations

CI = confidence interval; OR = odds ratio; VEGF = vascular endothelial growth factor.

## Competing interests

The authors declare that they have no competing interests.

## Authors' contributions

EJJ conceived the study, contributed to data analysis, and drafted the manuscript. HSF contributed to the study design and data analysis. EBB and KAB performed the statistical analyses. CR contributed to the study design, data collection, and data analysis. VLS contributed to data collection and data analysis. AVP contributed to data analysis. MJT and EEC contributed to the study design. In addition, all authors contributed to critical revision of the manuscript, and read and approved the final manuscript.

## Figures and Tables

**Table 1:** Odds ratios for breast cancer incidence by *VEGF *genotype.

Polymorphism	Controls (*n *= 504)	All cases^a ^(*n *= 501)		*In situ *cases (*n *= 107)		Invasive cases (*n *= 380)	
	
	*n *(%)	*n *(%)	OR (95% CI)	*n *(%)	OR (95% CI)	*n *(%)	OR (95% CI)
-2578C/A (rs 699947)							
AA	130 (26.3 %)	114 (22.9%)	1.00 (ref.)	31 (29.0 %)	1.00 (ref.)	81 (21.4 %)	1.00 (ref.)
AC	236 (47.7%)	245 (49.2%)	1.18 (0.86–1.61)	53 (49.5%)	0.92 (0.55–1.52)	184 (48.7%)	1.24 (0.88–1.74)
CC	129 (26.1%)	139 (27.9%)	1.25 (0.88–1.77)	23 (21.5%)	0.68 (0.37–1.24)	113 (29.9%)	1.46 (1.00–2.14)
*P *(for trend)			0.21		0.21		0.049
-1154G/A (rs 1570360)							
AA	57 (11.6 %)	51 (10.3%)	1.00 (ref.)	17 (16.0%)	1.00 (ref.)	33 (8.8%)	1.00 (ref.)
AG	227 (46.1%)	208 (42.0%)	1.01 (0.66–1.55)	48 (45.3%)	0.66 (0.34–1.25)	152 (40.5%)	1.14 (0.70–1.84)
GG	208 (42.3%)	236 (47.7%)	1.29 (0.84–1.97)	41 (38.7%)	0.62 (0.32–1.20)	190 (50.7%)	1.64 (1.02–2.64)
*P *(for trend)			0.083		0.24		0.007
-634G/C (rs 201963)							
GG	232 (46.4 %)	221 (44.7%)	1.00 (ref.)	56 (52.8%)	1.00 (ref.)	161 (42.9%)	1.00 (ref.)
CG	221 (44.2%)	222 (44.9%)	1.05 (0.80–1.37)	39 (36.8%)	0.73 (0.46–1.15)	174 (46.4%)	1.13 (0.85–1.50)
CC	47 (9.4%)	52 (10.5%)	1.18 (0.76–1.83)	11 (10.4%)	0.88 (0.42–1.84)	40 (10.7%)	1.27 (0.79–2.05)
*P *(for trend)			0.47		0.35		0.26
+936C/T (rs 3025039)							
TT or CT^b^	126 (26.3%)	128 (26.2%)	1.00 (ref.)	39 (38.2%)	1.00 (ref.)	86 (23.1%)	1.00 (ref.)
CC	353 (73.7%)	360 (73.8%)	1.02 (0.76–1.36)	63 (61.8%)	0.59 (0.37–0.93)	286 (76.9%)	1.21 (0.88–1.67)
*P *(for trend)			0.73		0.052		0.20
Blood prediction score^c^							
0–1	31 (6.5 %)	38 (7.8 %)	1.00 (ref.)	11 (10.9 %)	1.00 (ref.)	27 (7.3 %)	1.00 (ref.)
2	157 (33.0%)	131 (27.0%)	0.72 (0.42–1.22)	38 (37.6%)	0.75 (0.34–1.64)	90 (24.3%)	0.68 (0.38–1.22)
3	198 (41.6%)	214 (44.1%)	0.92 (0.55–1.55)	39 (38.6%)	0.56 (0.25–1.24)	166 (44.7%)	0.99 (0.57–1.75)
4	90 (18.9%)	102 (21.0%)	0.99 (0.57–1.72)	13 (12.9%)	0.39 (0.15–0.98)	88 (23.7%)	1.22 (0.67–2.23)
Trend OR (95% CI)^d^			1.09 (0.94–1.26)		0.73 (0.57–0.96)		1.20 (1.02–1.41)
*P *(for trend)			0.28		0.021		0.030

Odds ratios (ORs) are adjusted for birth-year, year of blood draw, and race/ethnicity. ^a^Includes 14 cases of unknown stage. ^b^The TT genotype was carried by eight controls and six cases (two *in situ *cases and four invasive cases). ^c^Sum of -2578C and +936C alleles, which have each been associated with increased blood levels of vascular endothelial growth factor. ^d^OR associated with a 1-point increase in the blood prediction score. CI, confidence interval;

**Table 2:** Odds ratios for invasive breast cancer incidence by *VEGF *promoter haplotype.

Haplotype copies	Cases/controls	OR (95% CI)
AAG		
0	188/204	1.0 (ref.)
1	149/224	0.69 (0.51–0.92)
2	33/56	0.62 (0.38–1.00)
*P *(for trend)		0.008
AGG		
0	260/342	1.0 (ref.)
1	98/133	0.97 (0.71–1.33)
2	12/9	1.71 (0.70–4.15)
*P *(for trend)		0.63
CGG		
0	230/327	1.0 (ref.)
1	125/144	1.27 (0.94–1.72)
2	15/13	1.68 (0.78–3.61)
*P *(for trend)		0.054
CGC		
0	161/220	1.0 (ref.)
1	170/217	1.07 (0.80–1.43)
2	39/47	1.18 (0.73–1.90)
*P *(for trend)		0.47

Haplotypes defined by polymorphisms at -2578, -1154, and -634 of the vascular endothelial growth factor gene. Includes 370 cases of invasive breast cancer and 484 controls with complete data on all three polymorphisms. Odds ratios (ORs) adjusted for birth year, year of blood draw, and race/ethnicity. CI, confidence interval.
